# Psychosocial Correlates of Frailty in Older Adults

**DOI:** 10.3390/geriatrics1040026

**Published:** 2016-11-01

**Authors:** Simone Freitag, Silke Schmidt

**Affiliations:** Department Health and Prevention, Institute of Psychology, Ernst-Moritz-Arndt-University Greifswald, Robert-Blum-Str. 13, 17487 Greifswald, Germany; silke.schmidt@uni-greifswald.de

**Keywords:** psychosocial correlates, frailty, older adults, gender differences, quality of life, depression, resilience, traumatic life events

## Abstract

Background: The aim of this study was to investigate psychosocial variables associated with frailty status. Moreover, gender differences in the psychosocial variables associated with frailty were examined. Methods: This cross-sectional study examined a community-dwelling sample of N = 210 older adults (*M* = 75.31 years). Frailty was measured with the Tilburg Frailty Indicator. Quality of life, depression, resilience, social support, self-efficacy, traumata experienced, and trauma severity were assessed as psychosocial variables. Logistic regression analyses were performed. Results: In total, 41.4% of the participants were frail (49.6% women, 27.8% men). Main correlates of frailty were depressive symptoms, quality of life, and resilience. Gender differences for frailty correlates were found. Depressive symptoms and perceived social support were mainly associated with frailty in women. Furthermore, age was only significantly associated with frailty in women. Quality of life was significantly associated with frailty being a protective factor in both women and men. Trauma count and trauma severity were associated with an elevated risk for frailty in men. Conclusions: The results show that the psychosocial variables depressive symptoms, quality of life, and resilience are associated with frailty. Gender-specific differences in psychosocial correlates of frailty were revealed. Results suggest that gender-specific assessments and interventions should be developed to prevent frailty in late life.

## 1. Introduction

Frailty will be an important public health care issue in future decades, especially in light of the growing proportion of older adults in western societies by 2050. It is associated with an increase in adverse outcomes such as disability, increased health care utilization, and mortality [1]. According to the conceptual framework of Gobbens et al. [1], frailty is a syndrome that affects biological, psychological, and social processes of a person’s life and leads to increased vulnerability and adverse outcomes in old age. Thus, people tend to decrease from a fit and healthy status to physical weakness and frailty when growing old [2]. Whereas the biological variables for frailty such as weight loss, imbalance, and hand strength are well examined, the literature lacks investigations of the influence of the psychological and social contributors of frailty [3]. Evidence shows that frailty is not only based on biomedical changes and physical deterioration but is also linked to psychological and social variables [2,4,5]. The causes of frailty are manifold, and the investigation of its associated contributors is ongoing. Likewise, despite the fact that women report more frailty symptoms than men, not much is known about gender-specific psychosocial correlates of frailty. Therefore, this study aims to address this research gap and investigates which psychosocial variables are associated with frailty status in older adults. Additionally, gender differences in psychosocial variables associated with frailty are examined more closely.

### 1.1. Psychosocial Correlates of Frailty

Research on psychosocial variables and frailty has shown that psychological disorders such as depression have an impact on frailty in older adults. Therefore, frailty is highly positively associated with depression [6], and depressive symptoms are part of the frailty assessment [7]. Despite this high correlation, findings show that only one quarter of depressed subjects was physically frail, especially those with high levels of depressive symptoms [8]. Hence, it can be expected that depressive symptoms are one key correlated for frailty status. Therefore, depression proved to be an important psychological correlate of frailty, but evidence is insufficient on other psychosocial variables and their influence on frailty. However, few studies have investigated other psychosocial correlates of frailty. A Dutch study found that high anxiety levels as well as low levels of wellbeing, sense of control, social activities, and home/neighborhood satisfaction were associated with frailty and with an increased likelihood for adverse outcomes [9].

The link between frailty and quality of life has been mainly investigated with respect to how frailty influences quality of life. Findings show that frailty and quality of life are negatively correlated [10]. Frailty and well-being also show a significant negative inter-correlation of *r* = −0.58, which was persistent even when controlling for age, gender, health behavior, and depressive symptoms [11]. Furthermore, a study showed five out of seven quality of life domains to be negatively associated with frailty. Only social relationships and participation have not been negatively affected by frailty [12]. A study investigating psychosocial resources on physical frailty also showed that higher levels of mastery and self-efficacy were associated with decreased odds of functional decline. Hence, psychosocial variables served as buffers against functional decline and mortality in frail older adults [13].

Considering a life course perspective, it is also relevant to take resilience and traumatic life events into account as contributing factors for frailty. Resilience is defined as the capacity to adapt to changing environmental challenges. Thus, Kuh and Network [14] raised the question of how social and psychological resilience is associated with frailty. Frailty is also often described as the loss of resilience in older adults [15]. As a matter of fact, no study has actually examined frailty and resilience. It can be assumed that resilience is positively associated with frailty due to an adaptation to increased vulnerability or negatively associated due to a protective role of resilience.

Evidence shows that traumatic life events are associated with long-term effects on health and with early mortality in late life [16,17]. Therefore, it can be argued that frailty must be associated with traumatic life events. Correspondingly, in the conceptual model of frailty by Gobbens et al. [1] traumatic life events are described as part of sociodemographic variables that contribute to the development of diseases and frailty. Despite the empirical findings on health and theoretical links with frailty, the association of traumatic life events on frailty has not yet been investigated. It can be hypothesized that more traumatic life events and increased posttraumatic stress symptoms are negatively associated with frailty.

### 1.2. Gender Differences in Frailty

Research has shown that more women than men are screened as frail [18,19]. In a Dutch study, 30.1% of men and 47.4% of women were identified as frail by the Tilburg Frailty Indicator [20]. However, it is hardly investigated why more women compared with men are frail and pre-frail. One explanation approach uses profiles of functioning patterns, in which women have less desirable profiles and are therefore more at risk for physical frailty and psychological malfunctioning [21]. For a fact, women have a higher life expectancy and report more frailty symptoms. The reasons for this disparity of frailty are manifold, such as reporting bias, comorbidities with other chronic diseases, more depressive and anxiety disorders in women, as well as pathophysiological contributors (e.g., inflammation and child birth) [22]. It is also argued that women have a higher tolerance for frailty burden and greater physiological resources [22]. In contrast, evidence shows that men and women with higher levels of psychological well-being were less likely to become frail over a four-year follow-up period [23], hence confirming that psychological well-being may be a protective factor of frailty. Based on these findings, it can be hypothesized that gender differences in psychosocial variables associated with frailty status exist. Therefore, this study aims to investigate whether women and men differ in the psychosocial factors associated with frailty.

### 1.3. Aim of the Study

In summary, empirical evidence suggests that depression, quality of life, and social relationships will be important correlates of frailty status. As shortly summarized, anxiety, self-efficacy, resilience, and the experience and severity of traumatic life events may be associated with health in older adults and may also serve as psychosocial contributors to frailty. Hence, besides already known correlates of frailty, other psychological and social variables need more attention. Therefore, it is necessary to examine psychosocial variables that have additional associations with frailty. This is the first study to investigate multiple psychosocial correlates of frailty in a German sample. It is hypothesized that quality of life and depressive symptoms will be important predictors for frailty status. It furthermore explores which other psychological variables will incrementally contribute to frailty. In addition, due to the gender disparity in frailty symptoms, gender-specific psychosocial contributors of frailty are investigated. It is hypothesized that frailty is associated with different psychosocial variables for women and men.

## 2. Materials and Methods

### 2.1. Sample and Study Design

The study was conducted between February and December of 2012 in two cities in Northern Germany. Participants were selected from an existing cohort sample in an urban area and a community-dwelling sample in a rural area of Germany. Participants living in urban areas and coming from a subsample of a cohort study were recruited via postal addresses. A total of 248 people were contacted in the urban area, of which 137 (55.2%) responded to the questionnaire. People from the rural area were recruited through newspaper announcements and senior offices. Potential participants called the study team and were sent the questionnaire with a reply paid envelope. The sample is a convenience sample of older adults who agreed to partake in the study. Participants were asked to fill out a questionnaire assessing health-related constructs, such as frailty, quality of life, sense of coherence, resilience, self-efficacy, social support, depressive symptoms, trauma, and anxiety.

Prior to the data conduction, all participants were informed about the content and the purpose of the study, and informed consent was signed. Personal data obtained was handled anonymously and ethical approval was granted by the Medical Chamber of Hamburg and the ethics committee of the University Medicine Greifswald.

### 2.2. Measurements

*Frailty*. Frailty was assessed with the Tilburg Frailty Indicator (TFI), a self-report questionnaire with 15 items on three domains—physical, social, and psychological. Physical frailty is assessed with eight items, including sudden weight loss, difficulties in walking, balance, eyesight, and hearing impairment. Psychological frailty is measured with four items regarding cognition, depressive symptoms, anxiety, and coping. Social frailty is assessed with three items concerning living alone, social relationships, and social support. A sum score ranges from 0 to 15. Eleven of the items have the answer format yes/no. Four items have the answer format yes/no/sometimes (cognition, depressive symptoms, anxiety, social relations). The cut-off score of 5 and higher is associated with being classified as frail [7]. The psychometric properties of the TFI are good with α = 0.79; the individual domains range from α = 0.67–0.78 for a 1-year interval [7]. It was also demonstrated good convergent and divergent construct validity [20]. The German version of the TFI was used with acceptable psychometric properties [24].

### 2.3. Psychological Variables

*Depressive symptoms*. The depressive module of the Patient Health Questionnaire (PHQ-9; [25]) was applied to assess depressive symptoms. This self-report measurement screens for depressive symptoms based on the DSM-IV criteria [26]. Nine items are answered on a 4-point Likert-scale from 0 (not at all) to 3 (almost every day), with a maximum sum score of 27. The PHQ-9 shows good sensitivity (95%) and specificity (86%) [27]. The PHQ-9 shows a very good internal reliability with Cronbach’s α = 0.88–0.89 [27,28]. Cut-off scores below 5 indicate the absence of a depressive syndrome, scores of 5 to 10 indicate a mild depressive syndrome, and above 10 indicate a major depressive syndrome [25].

*Quality of life*. The European Health Interview Survey-Quality of Life (Eurohis-8) is a self-assessment instrument of generic global quality of life [29]. The eight items are derived from WHOQOL-100 [30] and measure psychological, physiological, social, and environmental factors (two items each) of perceived quality of life. They refer to the last two weeks and are rated on a 5-point Likert scale. An overall quality of life sum score represents the extent of subjectively perceived quality of life. It shows good internal consistency with Cronbach’s α = 0.80 [31].

*Social support*. The Social Support Questionnaire in its short version (F-Soz-U-K-14) with 14 items measures three contents: emotional support (eight items), practical support (three items), and social integration (four items) [32]. The instrument is used to assess perceived or anticipated support from the social environment. A sum score can be calculated [32]. The reliability is very good with Cronbach’s α = 0.94. The instrument is normed on a representative German sample [32].

*Anxiety*. The Geriatric Anxiety Inventory-Short Form is a self-report instrument measuring anxiety symptoms of older people with five items (GAI-SF; [33]). The Short Form has a cutoff value of three and is adequate to identify Generalized Anxiety Disorder (GAD) according to DSM-IV. Internal consistency of the short version is α = 0.81. The GAI was primarily developed for use in health care and is also of avail in epidemiological research on anxiety [33].

*Self-efficacy*. The general self-efficacy (SE) scale is a self-report measure that assesses the self-perceived ability to handle and cope with difficult life situations based on the concept of self-efficacy by Bandura [34]. The scale consists of 10 items, answered on a 4-point Likert-scale ranging from “absolutely agree” to “absolutely disagree,” with a sum score that ranges from 10 to 40. The higher the sum score, the more optimistic the self-efficacy. There are standardized scores for age (14–95 years) and gender that are derived from a representative survey [35]. The SE scale has good psychometric properties with an internal consistency of Cronbach’s α between 0.80 and 0.90 in German surveys [36]. The instrument also shows good convergent and divergent validity [37].

*Resilience*. A short form of the Resilience Scale (RS-25; [38]) was used with 11 items that load on a single factor, indicating only one global score for analysis [39]. Resilience is conceptualized as protective personality factor. Participants answer on a 7-point Likert scale if they agree or disagree with statements such as “When I make plans I follow through with them”; “I usually manage one way or another”; “I feel that I can handle many things at a time”. The German short version (RS-11) is used in this study for economic reasons and its good reliability (Cronbach’s α = 0.81). Normed values for a wide age range exist [40].

*Traumatic life events and trauma severity*. The Posttraumatic Stress Disorder Checklist–Civilian Version (PCL-C; [41]) was used to assess symptom severity of traumatic life events. It is a self-report rating scale consisting of 17 items which correspond to DSM‑IV symptoms of Posttraumatic Stress Disorder. The extent of posttraumatic symptoms in the past month using a 5-point Likert-scale (“not at all” to “extremely”) is rated. The PCL has excellent internal consistency (α = 0.94–0.97) [41,42]. Furthermore, to assess the number of traumata experienced, a list of 15 traumatic events was presented, and participants had to report whether they experienced the events or not. A trauma score was calculated by summarizing the events experienced.

### 2.4. Statistical Analysis

Statistical analysis were performed with SPSS 22.0. Descriptive statistics were performed by frequencies and means, with chi-squared tests and *t*-tests (Bonferroni adjusted). Effect size Cohens *d* was calculated with conventions for effect sizes by Cohen [43] (small *d* < 0.4, moderate *d* = 0.5–0.7, large *d* > 0.08). Correlations were performed to investigate the relationships between the variables. Logistic regression analyses were performed to predict frailty status. Frailty status as the criterion variable was dichotomized as non-frail (0), with a TFI cut-off of 4 and below, and frail (1), with a TFI cut-off score of 5 and higher. The logistic regression analysis was performed in two steps. To control for age and gender, both variables were introduced as a block first. Secondly, all psychological variables were entered to explain the maximal variance of the regression model. Logistic regression analysis with backward elimination based on the likelihood-ratio statistic was used. This analysis tests a model with all predictors and removes predictors without statistical influence. As a result, variables with the strongest association with frailty are extracted.

## 3. Results

### 3.1. Sample Characteristics

A sample of N = 210 people, mean (*M)* age = 75.3 (standard deviation (*SD)* = 5.74) years (age range 64–91 years), was recruited in Northern Germany. It comprised N = 131 (62.4%) females and N = 79 males (37.6%). In Table 1, the descriptive characteristics are displayed for the total sample as well as for men and women. Men were one year older than women, but without significant statistical difference in the mean age. Most participants were either married (55.7%) or widowed (28.6%). Family status was significantly different in women compared with men. More women were divorced or widowed, whereas most men were married. This also reflects in the partnership status, where more women lived without a partner. Of the sample, 123 participants were not frail and 87 were classified as frail, with a cutoff of TFI sum score >5. The mean sum score in the sample was *M(SD)* = 4.08(2.62). Significant gender differences were found for all three TFI sub-dimensions and the TFI sum score. Women reported more physical, psychological, and social symptoms than men. In total, women reported a higher frailty sums score than men.

### 3.2. Correlations of Frailty and Psychosocial Variables

Frailty and age were significantly positively correlated with *r* = 0.19, *p* = 0.004. The older the participant, the more frailty symptoms were reported. Overall, inter-correlations (Table 2) show that the variables were highly significantly correlated with each other. Frailty and quality of life were associated negatively with *r* = −0.56, *p* < 0.01, which indicates that frail people were less likely to experience a high quality of life. The highest correlation was recognized between frailty and depressive symptoms with *r* = 0.63, *p* < 0.01, and frailty and trauma severity *r* = 0.54, *p* < 0.01.

### 3.3. Association of Frailty with Psychosocial Variables

From the total sample, only N = 196 people completed all questionnaires; N = 14 participants had missing values and were excluded from the regression analysis. The logistic regression analysis aimed to investigate which psychological variables are associated with frailty status. Therefore, frailty status was the dichotomous criterion variable (frail vs. non-frail). Age and gender were controlled for in the first block entered in the analysis. The psychosocial variables were introduced into the regression model as predictors in the second block (Table 3). Age and gender were significant predictors for frailty, and both predictors explained 12% of variance. The correct prediction was 58.2%. In the complete regression model, only age remains a significant predictor, indicating that increased age is accompanied by an increased likelihood for being frail. The stepwise introduction of the psychological variables showed three significant predictors for frailty: quality of life, resilience, and depressive symptoms. This regression model explained Nagelkerkes R^2^ = 58.8% of variance. This model correctly classified 84% of the participants, and 87% were correctly classified as non-frail, 79.7% were correctly classified as frail.

### 3.4. Gender Differences in Psychosocial Variables Associated with Frailty

Gender differences were evident for frailty status. Significantly more women (49.6%) than men (27.8%) were classified as frail, which was statistically significant (Chi-squared Х^2^ = 9.62, *p* = 0.002). Taking a closer look at the TFI sum score, it can be seen that women reported a higher sum score with M = 4.67 than men with M = 3.09, which is a statistically significant with an intermediate effect size (Х^2^ = 4.68, *p* < 0.001, Cohens *d* = 0.64). Compared with men, women also reported more symptoms in each TFI domain: physical (*d*_phys_ = 0.42), psychological (*d*_psy_ = 0.41), and social (*d*_soc_ = 0.54) with significant results and intermediate effect sizes according to Cohen [43].

The logistic regression analysis was also performed singularly for both women and men to explore whether there are gender differences in psychosocial contributors to frailty status. Therefore, gender as a predictor was removed in the first block.

*For women*. The logistic regression model showed that age remains a significant predictor for frailty status (Table 4). Additional significant predictors in the model were quality of life and depressive symptoms. Quality of life was shown to be a protective factor with a decreased likelihood for frailty. Depressive symptoms were significantly associated with an increased risk for frailty. Perceived social support was also extracted to be associated with frailty in women, but did not become significant. However, perceived social support can serve as a protective factor for women. The model explained Nagelkerkes R^2^ = 63.1% of the variance of frailty status. This model correctly classified 84.2% of the female participants, and 85.2% were correctly identified as non-frail and 83.1% were correctly identified as frail.

*For men*. The logistic regression analysis did not identify age as a significant predictor for frailty (Table 5). In contrast to women, the most important predictors for frailty in men were trauma severity, quality of life, resilience, and trauma count. However, only quality of life and resilience were statistically significant predictors. Both predictors were protective factors, meaning that men with high values on both variables were less likely to be frail. Even though trauma severity and trauma count did not become significant, it can be seen that both variables are accompanied by an increased likelihood for frailty. The model explained Nagelkerkes R^2^ = 65.1% of the variance of frailty status. This model classified 87.8% of male participants correctly, of which 94.4% were correctly identified as non-frail and 70% were correctly identified as frail.

## 4. Discussion

This study aimed to first investigate psychosocial correlates of frailty and secondly to examine gender-specific differences in psychosocial correlates of frailty. Therefore, based on evidence on the association of depressive symptoms and quality of life as correlates, additional psychosocial variables such resilience, self-efficacy, anxiety, traumatic life events, and trauma severity were investigated to explain incremental variance of frailty. In summary, the results showed that the main psychosocial frailty correlates in this sample were depressive symptoms, quality of life, and resilience. Moreover, gender-specific results emphasize different psychosocial correlates for frailty in men and women.

### 4.1. Psychosocial Correlates of Frailty

In fact, in the total sample age, depressive symptoms and quality of life were the main correlates for frailty, which is in accordance with our hypothesis. As a novel finding, resilience shown to be significantly associated with frailty. Participants with high levels of quality of life and resilience were less likely to be frail in this sample. Usually research shows that frailty is associated with poor quality of life in old age [10,44], but this may also be due to older samples with higher frailty levels. Therefore, quality of life and resilience may serve as protective factors because the odds for frailty were decreased in the presence of both variables. It has been argued that on a physical level a loss of resiliency leads to an increase of physical frailty [15]. Resilience as a psychosocial resource for frailty prevention has not yet been discussed in the literature. Hence, addressing resilience can also be valid in approaches to maintain good health and prevent frailty. Despite significant correlations with frailty, the variables self-efficacy, anxiety, social support, trauma count, and trauma severity did not become significantly associated with frailty in light of the other variables.

### 4.2. Gender Differences in Psychosocial Correlates of Frailty

Taking a closer look at the gender differences, depressive symptoms was a main predictor for women, but not for men. For women, higher levels of depressive symptoms were associated with frailty status. In both women and men, quality of life was a significant correlate for frailty, meaning that higher scores of quality of life were associated with lower levels of frailty. Furthermore, only age was a significant predictor for frailty in women. This might be due to the fact that women have a higher longevity and are therefore more likely to become frail, or as shown in other studies, women report more frailty symptoms than men [22]. Correspondingly, we were also able to show that 49.6% of the females were classified as frail compared to only 27.8% of the male participants. These results were similar to studies that found that in general men reported fewer symptoms than women [20]. The main risk factors for men were the experiences of more traumatic life events and trauma severity, which were both associated with an increased likelihood for frailty. This novel finding suggests the importance of life-course determinants and the influence of traumatic events on frailty. It also confirms the associations of traumatic life events proclaimed in the integral conceptual model of frailty [1]. Even though, resilience was a protective factor for men, meaning that higher resilience levels were associated with a lower likelihood for frailty. The novelty of these results are that psychosocial frailty correlates differ for men and women, which has not been shown before. These gender-specific protective and risk factors should be acknowledged in screenings and prevention strategies for frailty. Consequently, prevention programs for men and women should address gender-specific health aspects.

### 4.3. Limitations

Some limitations have to be mentioned. Despite the results, it is also possible that certain psychosocial variables lost their statistical influence in the presence of other variable combinations. Moreover, the results are also due to the high correlation of frailty and depressive symptoms, and depressive symptoms are also part of the frailty assessment. It can be argued that frailty and depressive symptoms share redundant variance, but they are based on different theoretical concepts. The same can be argued for the correlation of frailty and quality of life. Despite the construct overlap, there is also unexplained residual variance that cannot only be explained by the redundancy of variables. Another limitation lies within the sample characteristics, which included more fit individuals than frail people and could have caused biased results. However, it also gives insight into frailty onset and early detection. Moreover, more women than men took part in the study, which might have led to the specific results for gender. However, the gender distribution is common in community-dwelling samples of older adults. Furthermore, all variables were assessed by self-report, which may also account for a reporting bias in men and women. It is suggested that samples including more men must be investigated to confirm the results. The prevalence rates were quite high, with 41.1% in a community-based healthy sample. For once the prevalence rate using this instrument was higher than other frailty measures. However, this also raises the question of a low cut-off value or a high sensitivity of the TFI measurement. Researchers from Italy consider 6 instead of 5 to be a better TFI cut-off value [45]. Therefore, it can be assumed that a cut-off of 5 would lead to an over-report of frailty. The TFI is a very sensitive measurement and performs best in its psychometric properties compared with other frailty measures [46]. However, there is no gold standard for the assessment of frailty, and the combination of uni- and multidimensional frailty measures can be useful [5].

## 5. Conclusions

Frailty research needs to put emphasis on the psychosocial correlates of frailty. Future research should also examine the life-course determinants such as traumatic life experiences that increase the likelihood for frailty. The long-term impact of traumatic life events seems especially to have a crucial effect on frailty in men. In general, gender-differences should be acknowledged for frailty screening and prevention strategies. More women than men reach very old age, so prevention strategies need to be adapted accordingly. Interventions should include active engagement in social activities to reduce depressive symptoms and increase social support. In contrast, interventions for men should include reminiscence approaches to cope with traumatic experiences and to build resilience. Furthermore, research on the directionality of psychosocial variables to investigate predictors of frailty in longitudinal studies is encouraged.

## Figures and Tables

**Table 1:** Sociodemographic characteristics, frailty status and scores, gender differences.

	Total (N = 210)		Female (N = 131)		Male (N = 79)		Test Statistics	
	*N*	*%*	*N*	*%*	*N*	*%*	X^2^	*p*
Sex	210	100	131	62.4	79	37.6		
Age group								
<74 years	93	44.3	63	48.1	30	38	11.29	0.004 **
75–84 years	103	49	57	43.5	46	58.2		
>85 years	14	6.7	11	8.4	3	3.8		
Family status								
Single	10	4.8	8	6.2	2	2.5	9.85	0.02 *
Married	117	55.7	55	42.3	62	78.5		
Divorced	22	10.5	16	12.3	6	7.6		
Widowed	60	28.6	51	39.2	9	11.4		
Partnership								
Yes	107	51	52	40.6	55	72.4	9.05	0.003 **
No	97	46.2	76	59.4	21	27.6		
Frailty status								
Frail	87	41.4	65	49.6	22	27.8	9.62	0.002 **
Non-frail	123	58.5	66	50.4	57	72.1		
	*M*	*SD*	*M*	*SD*	*M*	*SD*	*t*	*p*
Age	75.31	5.74	74.97	6.14	75.89	4.98	−1.18	0.239
TFI physical domain	2.18	1.85	2.47	1.88	1.71	1.71	2.91	0.004 **
TFI psychological domain	0.88	0.92	1.02	0.96	0.65	0.80	2.92	0.004 **
TFI social domain	1.01	0.88	1.18	0.92	0.73	0.72	3.89	<0.001 **
TFI sum score	4.08	2.62	4.67	2.71	3.09	2.13	4.68	<0.001 **

Note: X^2^—Chi squared test statistic; *p*-value—probability of significance; * *p* < 0.05; ** *p* < 0.01.

**Table 2:** Intercorrelations of frailty and predictors (Pearson correlation coefficient).

	1	2	3	4	5	6	7	8	9	10
1 Frailty sum score	1									
2 Frailty status	0.840 **	1								
3 Quality of life	−0.562 **	−0.491 **	1							
4 PCL-C	0.545 **	0.511 **	−0.445 **	1						
5 Resilience	−0.429 **	−0.453 **	0.390 **	−0.404 **	1					
6 Social Support	−0.353 **	−0.337 **	0.372 **	−0.372 **	0.376 **	1				
7 Self-efficacy	−0.201 **	−0.212 **	0.200 **	−0.243 **	0.504 **	0.218 **	1			
8 Anxiety	0.478 **	0.410 **	−0.429 **	0.528 **	−0.317 **	−0.280 **	−0.331 **	1		
9 Trauma score	0.234 **	0.181 **	−0.064	0.202 **	−0.02	−0.016	−0.031	0.012	1	
10 Depressive symptoms	0.635 **	0.571 **	−0.529 **	0.739 **	−0.467 **	−0.428 **	−0.258 **	0.510 **	0.166 *	1

Note: Significance level * *p* < 0.05; ** *p* < 0.01.

**Table 3:** Logistic regression to predict frailty status in the total sample.

	*B*	*SE*	Wald	*df*	*p*	Exp(B)	CI for Exp(B)	
							Lower	Upper
Age	0.101	0.038	6.841	1	0.009	1.106	1.026	1.192
Gender	−0.719	0.441	2.661	1	0.103	0.487	0.205	1.156
Quality of life (Eurohis-8)	−0.181	0.061	8.788	1	0.003	0.835	0.741	0.941
Resilience	−0.071	0.026	7.252	1	0.007	0.932	0.885	0.981
Depressive symptoms	0.398	0.087	20.788	1	<0.001	1.489	1.255	1.767
Constant	1.259	3.721	0.114	1	0.735	3.522		

Note: N = 194, missing N = 14; *B*—regression coefficient; *SE*—standard error; Wald—Wald test statistic; *df*—degrees of freedom; *p*-value—probability of significance; CI—Confidence interval; Log-Likelihood = 151.24; Cox R^2^ = 0.436, Nagelkerkes R^2^ = 0.588.

**Table 4:** Logistic regression analysis to predict frailty status in women.

	*B*	*SE*	Wald	*df*	*p*	Exp(B)	CI for Exp(B)	
							Lower	Upper
Age	0.112	0.048	5.541	1	0.019	1.119	1.019	1.229
Quality of Life	−0.186	0.08	5.484	1	0.019	0.83	0.71	0.97
Perceived Social Support	−0.059	0.033	3.269	1	0.071	0.943	0.884	1.005
Depressive symptoms	0.567	0.127	20.062	1	<0.001	1.763	1.376	2.259
Constant	−2.034	4.612	0.195	1	0.659	0.131		

Note: N = 120, missing N = 11; *B*—regression coefficient; *SE*—standard error; Wald—Wald test statistic; *df*—degrees of freedom; *p*-value—probability of significance; CI—Confidence interval; Log-Likelihood = 89.36; Cox R^2^ = 0.473, Nagelkerkes R^2^ = 0.631.

**Table 5:** Logistic regression analysis to predict frailty status in men.

	*B*	SE	Wald	*df*	*p*	Exp(B)	CI for Exp(B)	
							Lower	Upper
Age	0.035	0.078	0.2	1	0.655	1.036	0.888	1.208
Trauma (PCL-C)	0.09	0.054	2.73	1	0.098	1.094	0.983	1.216
Quality of Life (Eurohis8)	−0.313	0.123	6.464	1	0.011	0.731	0.574	0.931
Resilience	−0.248	0.083	8.803	1	0.003	0.781	0.663	0.919
Trauma score	0.509	0.275	3.418	1	0.065	1.664	0.97	2.855
Constant	16.576	8.74	3.597	1	0.058	15,809,737.5		

Note: N = 74, missing 5; *B*—regression coefficient; *SE*—standard error; Wald—Wald test statistic; *df*—degrees of freedom; *p*-value—probability of significance; CI—Confidence interval; Log-Likelihood = 42.10; Cox R^2^ = 0.45, Nagelkerkes R^2^ = 0.654.
